# Deep-blue organic light-emitting diodes based on a doublet *d*–*f* transition cerium(III) complex with 100% exciton utilization efficiency

**DOI:** 10.1038/s41377-020-00395-4

**Published:** 2020-09-08

**Authors:** Liding Wang, Zifeng Zhao, Ge Zhan, Huayi Fang, Hannan Yang, Tianyu Huang, Yuewei Zhang, Nan Jiang, Lian Duan, Zhiwei Liu, Zuqiang Bian, Zhenghong Lu, Chunhui Huang

**Affiliations:** 1 Beijing National Laboratory for Molecular Sciences (BNLMS), State Key Laboratory of Rare Earth Materials Chemistry and Applications, Beijing Engineering Technology Research Centre of Active Display, College of Chemistry and Molecular Engineering, Peking University, 100871 Beijing, China; 2 Tianjin Key Lab for Rare Earth Materials and Applications, School of Materials Science and Engineering, Nankai University, 300350 Tianjin, China; 3 Department of Physics, Yunnan University, 2 Cuihu Bei Lu, 650091 Kunming, China; 4 Key Lab of Organic Optoelectronics and Molecular Engineering of Ministry of Education, Department of Chemistry, Tsinghua University, 100084 Beijing, China

**Keywords:** Organic LEDs, Optoelectronic devices and components

## Abstract

Compared to red and green organic light-emitting diodes (OLEDs), blue OLEDs are still the bottleneck due to the lack of efficient emitters with simultaneous high exciton utilization efficiency (EUE) and short excited-state lifetime. Different from the fluorescence, phosphorescence, thermally activated delayed fluorescence (TADF), and organic radical materials traditionally used in OLEDs, we demonstrate herein a new type of emitter, cerium(III) complex **Ce-1** with spin-allowed and parity-allowed *d*–*f* transition of the centre Ce^3+^ ion. The compound exhibits a high EUE up to 100% in OLEDs and a short excited-state lifetime of 42 ns, which is considerably faster than that achieved in efficient phosphorescence and TADF emitters. The optimized OLEDs show an average maximum external quantum efficiency (EQE) of 12.4% and Commission Internationale de L’Eclairage (CIE) coordinates of (0.146, 0.078).

## OLEDs: deep blue emission from cerium(III) complex

A luminescent cerium(III) complex could be promising for realizing efficient and stable deep-blue organic-LEDs required for display and lighting applications, arising from its theoretical high exciton utilization efficiency and short excited-state lifetime. Liding Wang, Zifeng Zhao, and coworkers from China have shown that a new cerium (III) complex called **Ce-1**, which supports a d-f energy level transition, can efficiently emit blue light with a high photoluminescence quantum yield up to 93% and a short excited-state lifetime of 42 ns. OLEDs fabricated from the complex offered a maximum external quantum efficiency of 12.4% and a luminance of about 1000 cd m^−2^, and their emission is characterized as being deep blue with CIE colour coordinates of (0.146, 0.078).

## Introduction

Organic light-emitting diodes (OLEDs) have been successfully commercialized in the niche display market and are now under intense research for other applications, such as solid-state lighting. During the past three decades, fluorescence^1^, phosphorescence^2–5^, thermally activated delayed fluorescence (TADF)^6–8^, and organic radical^9–11^ materials have been subsequently applied as emitters because of their high efficiency, long-term stability, and low cost. As a new type of emitter in OLEDs, cerium(III) complexes have many potential advantages. First, we propose that the theoretical exciton utilization efficiency (EUE) could be as high as 100%, since the cerium(III) complex shows a doublet 5*d*–4*f* transition of the single electron of the centre Ce^3+^ (4*f*^1^ configuration) ion rather than a singlet and/or triplet transition, which will not be limited by spin statistics^10,11^. Second, cerium(III) complexes are expected to be more stable in OLEDs since their excited-state lifetimes are generally tens of nanoseconds^12–15^. Third, cerium(III) complexes are inherent blue or ultraviolet emitters, as demonstrated in the literature, although their emission colours could be theoretically affected by the ligand field^16^. Moreover, cerium(III) complexes are inexpensive because the abundance of cerium in Earth’s crust is 0.006 wt%, which is four orders of magnitude higher than that of iridium (0.0000001 wt%) and even slightly higher than that of copper (0.005 wt%)^17^.

However, most reported cerium(III) complexes are non-emissive because classic ligands and solvent molecules are found to quench Ce^3+^ ion luminescence upon coordination^18^. Hence, electroluminescence (EL) studies on cerium(III) complexes are very rare, and their advantages have not been demonstrated. To date, there are only three examples of EL study of cerium(III) complexes in the literature^19–21^. Among these examples, the maximum external quantum efficiency (EQE) of the best result is below 1%. As a breakthrough, we report herein a novel and neutral cerium(III) complex **Ce-1** with rigid scorpionate ligands showing a high photoluminescence quantum yield (PLQY) up to 93% in doped film and consequently a high average EQE of 12.4% in prototype OLEDs.

## Results

### Synthesis and structure

**Fig. 1:**
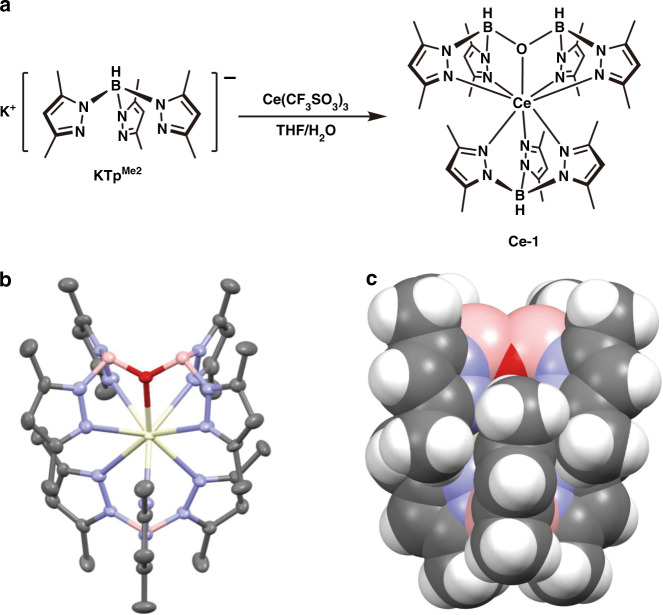
Synthesis and structure of the investigated cerium(III) complex Ce-1. **a** Synthetic route for the complex. **b** Single crystal structure of the complex shown as ellipsoids at the 50% probability level, where yellow represents Ce, pink represents B, blue represents N, red represents O, grey represents C, and the hydrogens are omitted for clarity. **c** Single crystal structure of the complex shown in a space-fill style, where hydrogens are shown in white

The complex **Ce-1** was synthesized by stirring potassium hydrotris(3,5-dimethylpyrazolyl)borate (KTp^Me2^)^22^ with Ce(CF_3_SO_3_)_3_ in tetrahydrofuran (THF), accompanied by hydrolysis due to a trace amount of water in the solvent (Fig. 1a). Though the reaction was found accidentally, it is repeatable and stable by adopting the conditions presented in the experimental section. Intriguingly, when KTp^Me2^ was replaced by potassium trispyrazolylborate (KTp) in this reaction, the expected complex Ce(Tp)_3_ was successfully obtained. The molecular structure and photoluminescence (PL) spectrum are exhibited in Supplementary Fig. S1. These results indicate that the methyl group on the pyrazole group has a crucial influence on the reaction. Most likely, the huge steric hindrance of the methyl group makes Ce(Tp^Me2^)_3_ unstable and tend to react with trace amounts of water in the solvent to form hydrolysate **Ce-1**.

The thermal stability of **Ce-1** is favourable, showing a decomposition temperature (*T*_d_, corresponding to a 1% weight loss) of 265 °C (Supplementary Fig. S2). Hence, the complex **Ce-1** was purified by thermal gradient sublimation at 230 °C and 2 × 10^−4^ Pa, during which single crystals were collected. The sublimated **Ce-1** was identified by high-resolution mass spectrometry, elemental analysis, and single crystal X-ray diffraction. No ^1^H NMR data were collected since **Ce-1** is paramagnetic, as demonstrated by electron paramagnetic resonance (EPR) spectroscopy (Supplementary Fig. S3). There are two almost identical coordination environments for Ce^3+^ ions in the crystal. The central Ce^3+^ ions are coordinated by tridentate and pentadentate ligands with one and two negative charges, respectively. There are seven nitrogen atoms and one oxygen atom surrounding cerium in **Ce-1**, with Ce–N distance in the range of 2.573–2.680 Å and a Ce–O distance of ~2.399 Å (Supplementary Table S1). Figure 1b, c shows one of the structures in ellipsoid and space-fill styles. From the space-fill model, it is found that the ligands shield the Ce^3+^ ion completely, which is important for high PL efficiency, since coordinating solvent molecules are frequently efficient quenchers of the lanthanide luminescence^23^.

### Photophysical properties

**Fig. 2:**
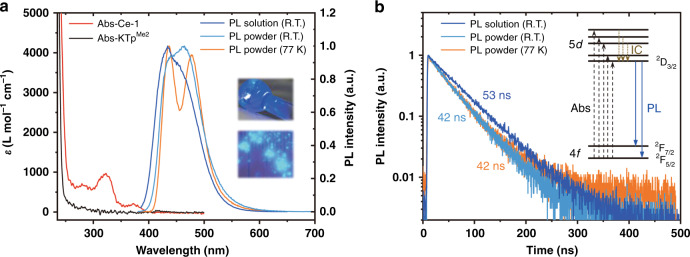
Photophysical properties of Ce-1 in solution and as crystalline powder. **a** UV–Vis absorption spectra of **Ce-1** and KTp^Me2^ in solution (DCM, 10^−5^ M), and PL spectra of **Ce-1** in solution (DCM, 10^−5^ M) and as crystalline powder at room temperature (RT) and 77 K. The excitation wavelength is 370 nm. Inset, photographs of the complex in solution and as crystalline powder under 365 nm irradiation. **b** Transient PL decays of the complex in solution and as crystalline powder at room temperature and 77 K. The excitation wavelength is 375 nm. Inset, schematic diagram to illustrate the PL mechanism of the cerium(III) complex, where Abs, IC, and PL represent absorption, internal conversion, and photoluminescence, respectively. ^2^D_3/2_ is the lowest excited state of the Ce^3+^ ion. ^2^F_5/2_ and ^2^F_7/2_ are two ground levels of the Ce^3+^ ion

Figure 2a shows UV–Vis absorption spectra of **Ce-1** and KTp^Me2^ in DCM solution. The three bands at 373, 322, and 280 nm with a molar extinction coefficient (*ε*) of ~10^2^ L mol^−1^ cm^−1^ can be assigned to the 4*f*–5*d* transitions of the Ce^3+^ ion by comparing the absorption spectra of **Ce-1** and KTp^Me2^. Another absorption band below 260 nm originates from the π–π* transition of the ligand. The excitation spectrum of **Ce-1** in DCM (Supplementary Fig. S4) has a similar pattern to its absorption spectrum. The peak at ~240 nm is attributed to ligand absorption, exhibiting cascade-type energy transfer from the ligand to the central Ce^3+^ ion under high-energy excitation. Due to parity-allowed characteristics, the complex can also be easily excited by direct *f*–*d* absorption (the peak at ~375 nm), while *f*–*f* excitation is difficult in a dilute solution of a typical Eu(III) complex due to the parity-forbidden characteristics of *f*–*f* absorption.

The dilute DCM solution and crystalline powder of **Ce-1** both exhibit strong blue emission. Their PL spectra (Fig. 2a) show two broad bands with different relative amplitudes. The redshift can be ascribed to molecule packing in crystals, while the relative amplitude may be sensitive to the coordination environment^24^. At 77 K, the emission spectrum of the crystalline powder splits into two peaks at 436 and 477 nm. The energy difference between the two peaks is close to 2000 cm^−1^, in agreement with the energy splitting between ^2^F_5/2_ and ^2^F_7/2_, two ground levels of the Ce^3+^ ion^20^. Moreover, the decay lifetimes of the solution at room temperature, crystalline powder at room temperature and crystalline powder at 77 K were measured to be approximately 53, 42, and 42 ns (Fig. 2b), respectively, which are comparable with those of previously reported cerium(III) complexes^12–14^. In addition, the emissions of **Ce-1** in solvents with different polarities showed almost identical spectra (Supplementary Fig. S5). This illustrates that unlike the famous radical emitter TTM-3NCz^11^, the electron transition in **Ce-1** is highly localized.

The PLQYs of **Ce-1** in dichloromethane (DCM, 10^−5^ M) and as crystalline powder are 48% and 82%, respectively. It should be noted that the PLQYs of **Ce-1** in DCM can be promoted by increasing the concentration, reaching ~100% at 10^−3^ M (Supplementary Table S2). By combining the lifetime and PLQY values, the radiative and nonradiative decay rates (*k*_r_ and *k*_nr_) are calculated as 9.1 × 10^6^ and 9.8 × 10^6^ s^−1^ for the DCM solution at 10^−5^ M and as 2.0 × 10^7^ and 4.3 × 10^6^ s^−1^ for the crystalline powder, respectively. The higher PLQY in the solid state is attributed to both an increased radiative rate constant and a decreased nonradiative rate constant, indicating that rigid molecular packing is beneficial for promoting radiative transition and inhibiting nonradiative transition. In addition, these values are comparable to those of organic radical materials and an order of magnitude larger than those of phosphorescence and TADF materials.

To obtain more in-depth insights into the excited state of **Ce-1**, theoretical calculations were carried out (Supplementary Fig. S6). The donor and acceptor for the first symmetry allowed transition were recognized as the 4*f* and 5*d*_*z*_^2^ orbitals, respectively. This result further proves the transition mechanism of **Ce-1**. Therefore, the observed strong blue emission can be attributed to the Ce^3+^ ion, more specifically to the two electric-dipole 5*d*–4*f* transitions of the Ce^3+^ ion from the lowest excited state (^2^D_3/2_) to the ground state ^2^F_5/2_ and ^2^F_7/2_, as shown in the inset of Fig. 2b.

### EL properties

The **Ce-1** complex shows high PLQY, excellent thermal stability, good stability in air (Supplementary Fig. S7), and short excited-state lifetime; it is worth investigating as the emitter in OLEDs with a vacuum thermal deposition method. The frontier molecular orbital energy levels of **Ce-1** were obtained as −6.2 eV for the highest occupied molecular orbital (HOMO) and −3.1 eV for the lowest unoccupied molecular orbital (LUMO) by investigating the ultraviolet photoelectron spectrum (Supplementary Fig. S8) and UV–Vis absorption spectrum. To optimize the OLED structure, the theoretical equation for calculating the EQE of OLEDs should be considered

EQE = ŋ_e–h_ × ŋ_PL_ × ŋ_out_ (1)

where ŋ_e–h_ is the recombination efficiency of injected holes and electrons, ŋ_PL_ is the intrinsic PL efficiency, i.e., the PLQY of the emission layer, ŋ_exciton_ is the EUE, i.e., the ratio of radiative excitons to total formed excitons, and ŋ_out_ is the light out-coupling efficiency. Based on the equation, the key is to screen host and carrier transport materials, hence achieving high PLQY in thin **Ce-1** film, and to balance and confine hole and electron recombination within/near the emission layer.

**Fig. 3:**
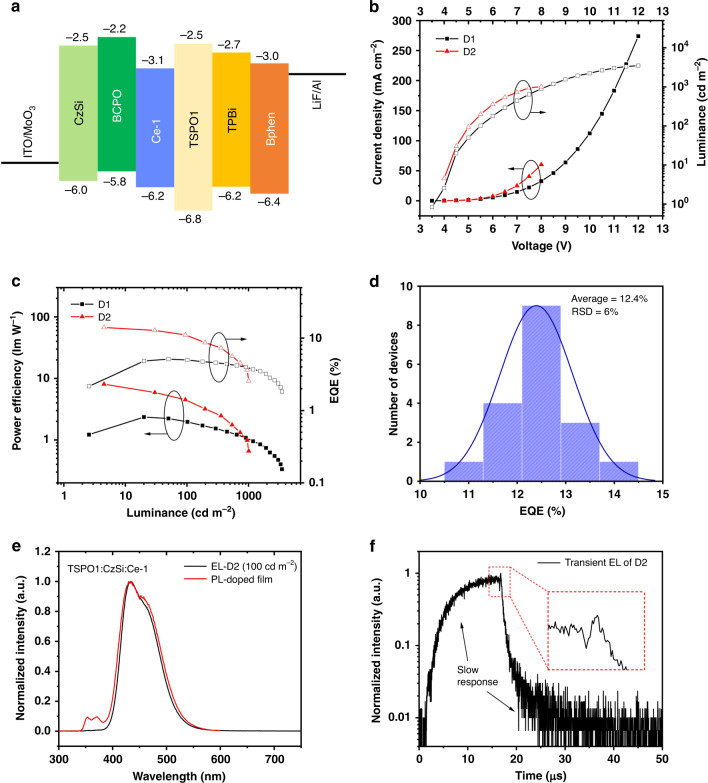
OLED demonstration. **a** Frontier orbital energy levels for materials used in OLEDs; the energy levels of **Ce-1** were deduced from its ultraviolet photoelectron spectrum and ultraviolet absorption spectrum. **b** Current density–voltage–luminance traces for the key OLEDs. **c** Power efficiency–luminance–EQE traces for the key OLEDs. **d** Maximum EQE histogram of 18 devices with the optimized device structure (average, ~12.4%; highest, 14.0%). **e** PL spectrum of the TSPO1:CzSi:**Ce-1** (0.18:0.72:0.1 in weight ratio, excitation 280 nm) film, and EL spectrum of **D2** at the luminance of 100 cd m^−2^. **f** Transient EL spectrum of **D2**

First, bis[4-(N-carbazolyl)-phenyl]phenylphosphine oxide (BCPO) was chosen as the host due to its high triplet energy level and excellent bipolar carrier mobility. Device **D1** was fabricated with a structure of indium tin oxide (ITO)/MoO_3_ (2 nm)/MoO_3_-doped 9-(4-tert-butylphenyl)-3,6-bis(triphenylsilyl)-9*H*-carbazole (CzSi:MoO_3_, 20 wt%, 10 nm)/CzSi (30 nm)/BCPO:**Ce-1** (10 wt%, 20 nm)/diphenyl-4-triphenylsilylphenyl-phosphine oxide (TSPO1, 10 nm)/2,2′,2′′-(1,3,5-benzinetriyl)-tris(1-phenyl-1-*H*-benzimidazole) (TPBi, 40 nm)/LiF (0.7 nm)/Al (100 nm). The frontier orbital energy levels of the utilized materials^25–27^ are shown in Fig. 3a. The device characteristics are plotted in Fig. 3b, c. The device emits deep-blue light similar to the **Ce-1** solution with CIE coordinates of approximately (0.15, 0.08) (Supplementary Fig. S9). Considering that the doped BCPO:**Ce-1** (10 wt%) film has a moderate PLQY of 26%, the device shows a relatively good maximum EQE of 5.1% and low efficiency roll-off (Fig. 3b). To estimate the EUE of **D1** more accurately, we used the variable-angle spectroscopic ellipsometry (VASE) method to determine the molecular orientation distribution in the emission layer (Supplementary Fig. S10 and Table S3)^28^. The **Ce-1** molecules show a horizontal dipole ratio of 68.1% in the BCPO film, which is close to the randomly oriented ratio (66.7%)^29,30^, indicating ~20% ŋ_out_, and hence, an ~100% EUE is demonstrated in **D1**. Thus, we expect that the performance of our deep-blue OLEDs could be further improved by screening the host material to obtain a higher PLQY.

Therefore, a series of **Ce-1**-doped films were fabricated in a vacuum chamber and subsequently measured for PLQYs and PL spectra (Supplementary Fig. S11 and Fig. 3e). Among these films, the TSPO1:**Ce-1** (10 wt%), CzSi:**Ce-1** (10 wt%), and TSPO1:CzSi:**Ce-1** (0.18:0.72:0.1 in weight ratio) films show relatively high PLQYs up to 70%, 83%, and 93%, respectively. With substantial device optimization (see Supplementary Table S4 for details), a champion performance was obtained for **D2** with the structure of ITO/MoO_3_ (2 nm)/CzSi:MoO_3_ (20 wt%, 30 nm)/CzSi (10 nm)/TSPO1:CzSi:**Ce-1** (0.18:0.72:0.1 in weight ratio, 20 nm)/TSPO1 (10 nm)/bathophenanthroline (Bphen, 40 nm)/LiF (0.7 nm)/Al (100 nm). The device characteristics are plotted in Fig. 3b, c for comparison. The champion performance includes a turn-on voltage of 3.6 V, a high maximum EQE of 14.0%, and a typical emission from **Ce-1** with CIE coordinates of (0.146, 0.078). This result is comparable to the best reported deep-blue OLEDs with a platinum complex, an iridium complex or TADF as the emitter (Supplementary Table S5)^31–33^. To estimate the EUE of **D2**, we measured the horizontal dipole ratio of **Ce-1** in TSPO1:CzSi (0.18:0.72 in weight ratio) as only 56.0% (Supplementary Fig. S10 and Table S3), which is even lower than that of the random orientation (66.7%), leading to an ŋ_out_ lower than 20%. Considering the PLQY of 93% and EQE of 14.0%, the EUE of **D2** is deduced to be higher than 70% (Table 1).

**Table 1:** Summarized parameters of key OLEDs with Ce-1 as the emitter

Device	Host	*V*_on_^a^ [V]	EQE_max_^b^ [%]	CE_max_^c^ [cd A^−1^]	*L*_max_^d^ [cd m^−2^]	CIE^e^
**D1**	BCPO					(0.148, 0.083)
	Average^f^	3.6	5.0	3.4	3457	
	Champion	3.6	5.1	3.5	3494	
**D2**	TSPO1:CzSi					(0.146, 0.078)
	Average^g^	3.8	12.4	8.7	948	
	Champion	3.6	14.0	10.3	1008	

^a^Turn-on voltage, taken as the reference point at which the luminance is 1 cd m^−2^

^b^Maximum EQE

^c^Maximum current efficiency

^d^Maximum luminance

^e^Coordinates at 100 cd m^−2^

^f^Average of three devices

^g^Average of 18 devices

**Fig. 4:**
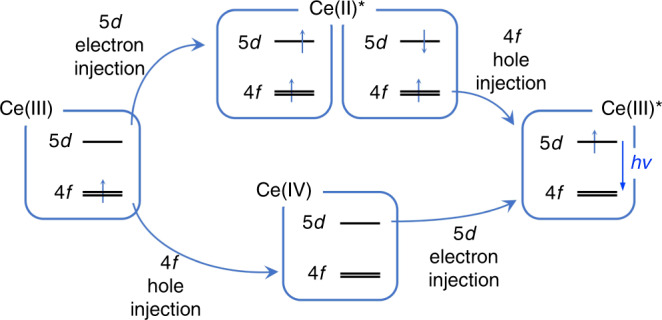
Speculated electroluminescence mechanism. The electron or hole is captured by a Ce(III) ion to form Ce(II)* (top route) or Ce(IV) (bottom route); then, hole or electron injection brings the intermediate species to the excited Ce(III)* ion state

The details of carrier recombination are critical for explaining the high EUE (100% for **D1** and >70% for **D2**) of these devices. As depicted in Fig. 3e, Supplementary Figs. S9 and S11, the steady-state PL spectra of emission layers contain deep-blue emissions that are substantially identical to the solution emission of **Ce-1** and weak ultraviolet emissions from host materials, indicating inefficient energy transfer from hosts to **Ce-1**. In contrast, the EL spectra of these devices exhibit pure emissions from **Ce-1**, implying that carrier recombination dominantly occurs on **Ce-1** rather than on host molecules. This inference can be further confirmed by the transient EL spectrum of **D2** (Fig. 3f). The spike (in the red dashed square) is proven to be evidence of charge trapping on the guest^34^. Interestingly, the response time of the EL intensity to the applied electric field is slow, implying that there may be a slow process of charge migration and carrier trapping in **D2**. After the polarity of the electric field is reversed, the remaining carriers in the device de-trap and continue to recombine on the guest, resulting in a microsecond delay that is much longer than the excited-state lifetime of **Ce-1**. Moreover, the absorption edge of the ligand is ~250 nm, corresponding to a bandgap of ~5 eV. However, the turn-on voltages of **D1** and **D2** are in the range of 3.6–3.8 V, which are much lower than the bandgap of the ligand. This implies that carriers recombine on the Ce^3+^ ions with a narrower bandgap instead of on the ligands. The speculated EL mechanism is exhibited in Fig. 4. Thus, doublet exciton formation is dominant in these OLEDs. Since Ce(III) ions are more likely to lose only one electron from the 4*f* orbital to form Ce(IV), the bottom route would be the main route for exciton formation in the device.

**Fig. 5:**
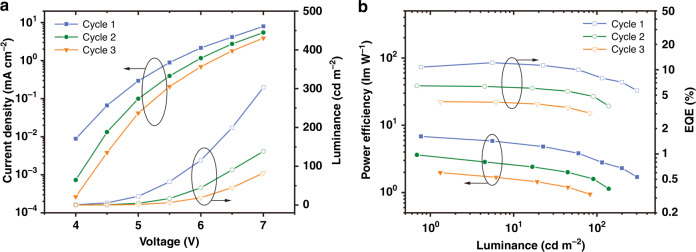
Cycling demonstration of device D2. **a** Current density–voltage-luminance traces. **b** Power efficiency–luminance–EQE traces

Although device **D2** shows high efficiency, the maximum luminance of **D2** is only 1008 cd m^−2^, and the efficiency roll-off is severe, which is contrary to the common knowledge that a short excited-state lifetime emitter is beneficial for reducing efficiency roll-off. In addition, the lifetime (LT_50_) of **D2** was measured as only 147 s (Supplementary Fig. S12). To obtain more in-depth insights into the device efficiency roll-off and stability, we measured device **D2** over three cycles and found that the device performance rapidly degrades (Fig. 5), indicating that the efficiency roll-off should be ascribed to the instability of the device. Furthermore, we introduced a florescence deep-blue emitter, 1-(10-(4-methoxyphenyl)anthracen-9-yl)-4-(10-(4-cyanophenyl)anthracen-9-yl)tetraphenylethene (TPEA)^35^, and a classic host material, 1,3-bis(N-carbazolyl)benzene, as references to fabricate control devices (Supplementary Fig. S12). Detailed results indicate that the instability of the host material, especially TSPO1^36–38^, is an important reason for device degradation. In addition, the Ce(III) complex is known to be a strong photo-reductant. This could be another reason for the device instability. Thus, the stability of **Ce-1** in terms of EL remains to be explored.

## Discussion

Materials with doublet emission from single-electron transition could endow OLEDs with ~100% EUE and a short excited-state lifetime (tens of nanoseconds, close to that of fluorescence materials). Organic radical emitters have achieved highly efficient doublet deep-red devices^11^. In this work, we have demonstrated a high EQE in deep-blue OLEDs based on a new cerium(III) complex **Ce-1** as the emitter, which shows parity-allowed doublet *d*–*f* transition of the centre Ce^3+^ ion with similar advantages in OLEDs. Interestingly, the organic radical emitter is beneficial for obtaining OLEDs with low-energy transitions, such as red and infra-red^39^, while the *d–f* transition-based Ce(III) complex is good for achieving OLEDs with high-energy transitions, such as blue and ultraviolet. Although the 147 s device lifetime is short, we believe this is a good start for deep-blue (CIEy < 0.1) devices, especially considering that no device lifetime of such deep-blue phosphorescence and TADF devices is currently reported.

## Materials and methods

### Materials

MoO_3_, BCPO, CzSi, TSPO1, TPBi, Bphen and LiF were purchased from Luminescence Technology Corp. TPEA was provided by the authors of literature^35^.

### Synthesis of Ce-1

KTp^Me2^ (2.02 g, 6 mmol), Ce(CF_3_SO_3_)_3_ (1.17 g, 2 mmol), H_2_O (0.036 g, 2 mmol), and dry THF (50 mL) were added to a 100 mL round-bottom flask. The mixture was stirred in a glovebox at room temperature for 3 days. After filtering off the insoluble part, the solvent was removed under vacuum, and the resulting solid was loaded into a thermal sublimator. With a gradient temperature of 230–150–80 °C and a pressure of ~2 × 10^−4^ Pa, 0.27 g **Ce-1** was obtained as crystalline powder in 14 h, with a yield of 15.8%. Anal. calcd. for **Ce-1**: N 22.87%; C 49.03%; H 6.11%; found: N 22.87%; C 48.83%; H 6.06%. MALDI-HR-ICRMS calcd. for **Ce-1** [C_35_H_52_B_3_CeN_14_O] 857.3782, found (M + H)^+^ 858.3835. Crystallographic data of **Ce-1** (CCDC 1913494).

### General characterization

Elemental analyses were performed on a VARIO EL analyser (GmbH, Hanau, Germany). High-resolution mass spectra were collected on a Bruker Solarix XR FTMS by the matrix-assisted laser desorption ionization (MALDI) method. UV–vis absorption spectra were recorded on a Shimadzu UV-3100 spectrometer. Fluorescence and transient PL decay spectra were measured on an Edinburgh Analytical Instruments FLS980 spectrophotometer. PLQYs were measured on a C9920-02 absolute quantum yield measurement system from Hamamatsu Company. Thermogravimetric analysis was undertaken with a Q600SDT instrument. Ultraviolet photoelectron spectroscopy was performed on an AXIS Supra X-ray photoelectron spectrometer.

### Density functional theory (DFT) calculations

All calculations were performed with the ORCA programme package^40^. For ground state geometry optimizations, the hybrid B3LYP^41–44^ density functional was used without symmetry constraints. The all-electron triple-ξ quality Def2-TZVP basis^45^ sets were assigned for B and N atoms. The Def2-ECP pseudopotential^46^ with Def2-TZVP valence basis sets was used for Ce (28 core electrons). Def2-SV(P) basis sets^47^ were applied for the remaining elements in these compounds. The RI plus chain of spheres (RIJCOSX for B3LYP) approximation^48^ was used to accelerate the calculations with Weigend’s “universal” Coulomb fitting auxiliary basis set def2/J^49^. We included the atom-pairwise dispersion correction with Becke–Johnson damping (D3BJ) to account for the van der Waals interaction^50–53^. In the single-point time-dependent DFT (TD-DFT) calculation, the B3LYP functional was applied with Def2-TZVP basis sets for all elements (the Def2-ECP pseudopotential was also applied for Ce).

### OLED fabrication and measurement

Commercially available ITO-patterned anodes with a sheet resistance of 14 Ω square^−1^ and an 80-nm thickness were used. ITO substrates were cleaned with deionized water and ethanol. The organic and metal layers were deposited in different vacuum chambers with a base pressure better than 1 × 10^−4^ Pa. The active area for each device was 4 mm^2^. All electric testing and optical measurements were performed under ambient conditions with encapsulation of devices in a glovebox. The EL spectra, current density–voltage–luminance (*J–V–L*) characteristics, EQE characteristics and device lifetimes were measured by a computer-controlled Keithley 2400 source meter and an absolute EQE measurement system (C9920-12) with a photonic multichannel analyser (PMA-12, Hamamatsu Photonics).

### VASE method

Doped films with a 20 nm thickness were deposited onto a clean quartz substrate for the VASE (ESM-300, J. A. Woollam Co.) measurement. The doped films were fabricated via thermal evaporation in a vacuum better than 1 × 10^−4^ Pa. The total deposition rate was ~1 Å s^−1^, and the doping concentration of **Ce-1** was 10 wt%, consistent with the emission layer in OLEDs.

### Transient EL measurement

Short-pulse excitation with a pulse width of 15 μs was generated using an Agilent 8114A. The amplitude of the pulse was 9 V, and the baseline was –3 V. The period was 50 μs, the delay time was 25 μs, and the duty cycle was 30%. The decay curves of the devices were detected using an Edinburgh FL920P transient spectrometer.

### EPR measurements

cw-EPR spectra were measured on a Bruker Elexsys E580 spectrometer with a superhigh sensitivity probehead (*f* = 9.3757 GHz). The low-temperature environment was achieved by an Oxford Instruments ESR900 liquid helium cryostat.

## Supplementary information

Supplementary information
