# Supplementary material for: Deep-blue organic light-emitting diodes based on a doublet d–f transition cerium(III) complex with 100% exciton utilization efficiency
Source: Light Sci Appl. 2020 Sep 8;9:157. doi: 10.1038/s41377-020-00395-4 (PMC7477100; doi:10.1038/s41377-020-00395-4)
Supplement: Supplementary file 1 — Supplementary information [file 41377_2020_395_MOESM1_ESM.docx]

Supplementary Information for

**Deep-blue** **organic light-emitting diodes based on a doublet d-f transition cerium(III) complex with 100% exciton utilization efficiency**

Liding Wang,^1†^ Zifeng Zhao,^1†^ Ge Zhan,^1^ Huayi Fang,^2^ Hannan Yang,^3^ Tianyu Huang,^4^ Yuewei Zhang,^4^ Nan Jiang,^3^ Lian Duan,^4^ Zhiwei Liu,^1*^ Zuqiang Bian,^1^ Zhenghong Lu,^3^ Chunhui Huang^1^

^1^Beijing National Laboratory for Molecular Sciences (BNLMS), State Key Laboratory of Rare Earth Materials Chemistry and Applications, Beijing Engineering Technology Research Centre of Active Display, College of Chemistry and Molecular Engineering, Peking University, Beijing, 100871, China.

^2^Department of Chemistry, Fudan University, Shanghai 200433, China

^3^Department of Physics, Yunnan University, 2 Cuihu Bei Lu, Kunming, 650091, P. R. China.

^4^Key Lab of Organic Optoelectronics and Molecular Engineering of Ministry of Education, Department of Chemistry, Tsinghua University, Beijing, 100084, China.

*****Corresponding author. Email: zwliu@pku.edu.cn

^†^These authors contributed equally to this work

Table of Contents

[Synthesis of Ce(Tp)_3_ 2](#_Toc49157467)

[Supplementary Fig. S1. The PL spectrum of Ce(Tp)_3_ powder. 2](#_Toc49157468)

[Supplementary Fig. S2. Thermogravimetric analysis of Ce-1. 2](#_Toc49157469)

[Supplementary Fig. S3. Electron paramagnetic resonance (EPR) spectrum of Ce-1 powder at 9.3 K. 3](#_Toc49157470)

[Supplementary Fig. S4. Excitation spectrum of Ce-1 in 10^-5^ M DCM solution. 3](#_Toc49157471)

[Supplementary Fig. S5. PL spectra of Ce-1 in DCM at different concentrations (left) and in varied solvents with different polarizability indexes (right). 3](#_Toc49157472)

[Supplementary Fig. S6. Theoretical calculated donor and acceptor natural transition orbitals (NTOs) of the first symmetry allowed transition. 4](#_Toc49157473)

[Supplementary Fig. S7. The PLQY variation of Ce-1 powder in the ambient environment. 7](#_Toc49157474)

[Supplementary Fig. S8. Ultraviolet photoelectron spectroscopy of Ce-1. 7](#_Toc49157475)

[Supplementary Fig. S9. Electroluminescence spectrum (black line) of device D1. 8](#_Toc49157476)

[Supplementary Fig. S10. Anisotropic refractive indices and extinction coefficient of Ce-1 doped films. 8](#_Toc49157477)

[Supplementary Fig. S11. Photoluminescence and electroluminescence spectra of Ce-1 doped films. 9](#_Toc49157478)

[Supplementary Fig. S12. Device operation lifetimes. 9](#_Toc49157479)

[Supplementary Table S1. Selected coordination bond lengths (Å) of Ce-1. 10](#_Toc49157480)

[Supplementary Table S2. The PLQYs of Ce-1 solutions. 10](#_Toc49157481)

[Supplementary Table S3. Molecular orientation measurements of Ce-1 doped films. 10](#_Toc49157482)

[Supplementary Table S4. The key parameters of OLEDs with Ce-1 as the emitter. 10](#_Toc49157483)

[Supplementary Table S5. The summary of deep-blue OLEDs. 13](#_Toc49157484)

[References 14](#_Toc49157485)

# Synthesis of Ce(Tp)_3_

Ce(CF_3_SO_3_)_3_ (0.6 mmol) and potassium trispyrazolylborate (1.9 mmol) were separately dissolved in 5 mL of ultra-dry methanol. After mixing the colorless solutions, a white powder was immediately precipitated. The mixture was stirred at room temperature for 18 h, and filtered to obtain a white powder, which was purified by thermal gradient sublimation. Total yield 82%. MALDI-TOF (m/z): calcd for C_27_H_30_B_3_CeN_18_ 779.2234, found 780.2329 (M+H)^+^. Anal. calcd for C_27_H_30_B_3_CeN_18_ (found): C 41.62% (41.47%), H 3.88% (3.80%), N 32.36% (32.33%).

# Supplementary Fig. S1. The PL spectrum of Ce(Tp)_3_ powder.

The excitation wavelength is 350 nm. Inset: the molecular structure of Ce(Tp)_3_.

# Supplementary Fig. S2. Thermogravimetric analysis of Ce-1.

# Supplementary Fig. S3. Electron paramagnetic resonance (EPR) spectrum of Ce-1 powder at 9.3 K.

# Supplementary Fig. S4. Excitation spectrum of Ce-1 in 10^-5^ M DCM solution.

The emission wavelength is 432 nm.

# Supplementary Fig. S5. PL spectra of Ce-1 in DCM at different concentrations (left) and in varied solvents with different polarizability indexes (right).

# **Supplementary Fig. S****6. Theoretical calculated donor and acceptor natural transition orbitals (NTOs) of the first symmetry allowed transition.**

The transition wavelength of 394 nm matches the absorption band of **Ce-1**.

**Coordinates from ORCA-job GS**

Ce 5.85912048226941 11.20125357578765 5.53701163268090

O 7.36540111862901 10.59476559339912 7.31984819422556

N 5.66720606019557 11.19510339602718 8.90414088623236

N 4.72913371745934 11.23758578143145 7.91340767350737

N 7.33962736970329 12.89670170093761 8.10249680094030

N 6.78676401781580 13.31039418764100 6.92987981522903

N 9.16814058528895 11.45408652897561 5.88939479488531

N 8.35326403248333 11.87126191080073 4.88170837607187

N 8.39089766394999 9.05734899264137 5.81597659297984

N 7.16487364118347 8.96270396249420 5.22823141153082

N 2.94231634210716 9.71050159526943 4.40558131114014

N 3.96069910090994 9.49557770751573 5.28356909338075

N 4.30141095936325 10.51422634222831 2.42332658194003

N 5.57156875394234 10.72188331521675 2.89492488512999

N 3.02655157831528 12.19956729472466 3.84320731741381

N 3.62849600493146 12.53980718061672 5.02023412549275

C 5.83985791659930 10.95122526264957 11.38884670497014

H 6.64882802132004 10.20931371414460 11.31654227803449

H 5.16426095286545 10.65446985670923 12.20353624322766

H 6.30630542270958 11.91084888368720 11.66598009370262

C 5.07686889286622 11.05176733988948 10.10742896444367

C 3.69919438509712 11.00863757216370 9.89134441789963

H 2.92354974513165 10.89289745874696 10.64520248769598

C 3.52842825273319 11.12364872314923 8.49850513232849

C 2.24975496771592 11.08333728859622 7.72407251921214

H 2.38090838658961 11.51413817518092 6.72534011491343

H 1.45943665486419 11.63685144748450 8.25363660875248

H 1.89937173455933 10.04744963469425 7.59109884226821

C 8.73370545574029 13.74801504112188 9.99177075635849

H 8.03667095287421 13.51407299759920 10.81194995833085

H 9.26613508574392 14.67454966873288 10.24936601759775

H 9.46487534080193 12.92584774846314 9.95140685906873

C 8.01442116741440 13.90630703958489 8.69023179757509

C 7.89251802405652 15.02411650325865 7.86488802568292

H 8.32548005576577 16.00719697839267 8.03448609522422

C 7.11761966925705 14.59491616052164 6.76806297911965

C 6.67941802106696 15.35809172533647 5.55960573034418

H 7.54143539399538 15.78738581030463 5.02635603310475

H 6.00923187296044 16.19148059205295 5.82621760546999

H 6.13549470189368 14.70514629685591 4.86145036045171

C 11.34854294545124 12.04397701279204 6.96447170034250

H 12.21568606081431 12.67153181259211 6.71667282461437

H 11.68674278167992 11.00206985289586 7.06148099683353

H 10.97007393282501 12.35666966999343 7.95114049950448

C 10.29143480461838 12.19972879245601 5.92160012396259

C 10.21347297207000 13.12111017703294 4.87430111544219

H 10.95565608968275 13.86891154645777 4.60281120913700

C 8.97660493461504 12.87267608436969 4.25090385781841

C 8.38951621679488 13.52064285104444 3.03681791502685

H 7.32120922735549 13.27966732489127 2.93095615131250

H 8.89767775445136 13.16595241304216 2.12512074489658

H 8.50275047140302 14.61500213343362 3.06837338781806

C 10.59102254198129 7.87646098116776 5.89249190561561

H 11.19411917216991 8.75943129147657 5.62478186590373

H 11.04701446568744 6.99826224077033 5.41508314021440

H 10.65876666246464 7.75284981902633 6.98544919025902

C 9.17516596936055 8.02904390559846 5.43877571967654

C 8.41996171459350 7.22049900757548 4.58666779171882

H 8.74094924692177 6.29981232273734 4.10512187408222

C 7.16326364977182 7.84661378746596 4.48625020602175

C 5.95801760940112 7.38275678794506 3.73036094335874

H 5.26206625275959 6.83249731743969 4.38493944599643

H 6.25431472707213 6.71024937859735 2.91279721034560

H 5.39929283769321 8.22402824412134 3.30821445029440

C 3.12073101008368 9.52422513724382 0.43845642967678

H 2.52551334664783 8.78490440496174 0.99606162058940

H 3.41619978474915 9.07306704806912 -0.51869591345272

H 2.45892749840736 10.37830188754623 0.22907301503469

C 4.33992802111138 9.93171605247999 1.20133271226807

C 5.67687659394349 9.77114700963251 0.85912909020665

H 6.07047466375319 9.32670548424827 -0.05197113226281

C 6.41288065252417 10.26586559643368 1.95268298029779

C 7.89783702412287 10.22232511719345 2.11082817586469

H 8.39874261129403 10.89686578316743 1.39863246110583

H 8.26220545078493 9.20353867488013 1.90682723670145

H 8.20600064158768 10.49597792341977 3.12361998621920

C 0.66518778750399 8.79487718835903 3.86066643437868

H 0.15581521393513 9.77003211636487 3.90845023623836

H -0.00739927821614 8.03652559541022 4.28497961481719

H 0.81957415643610 8.55751334501401 2.79583620289621

C 1.95014852796181 8.81301376920079 4.62309324650204

C 2.35547122484328 7.98744582466775 5.66999542482436

H 1.79239149041839 7.16683202216563 6.10790248474116

C 3.62620964844788 8.45702243572672 6.05482754179370

C 4.53462489725426 7.93900546051078 7.12387185277371

H 5.29934760067586 7.26619978582463 6.70340925678124

H 3.95904178187591 7.38241714995375 7.87749958319035

H 5.06916538750098 8.75439538672604 7.62831900996161

C 1.54100727731080 13.18503482317480 2.08425928844470

H 2.18786907238394 12.87127135481718 1.24992327364128

H 1.12714541022047 14.17454210005329 1.84539860970931

H 0.70486311367101 12.46998155771461 2.13665128087162

C 2.30209587636735 13.24070746765553 3.36949706603927

C 2.41764086946774 14.28167881953152 4.28707678616696

H 1.94772977435634 15.25992023844630 4.22267769772390

C 3.24122697191405 13.78941537005505 5.31850454793231

C 3.56872783748894 14.42202233135488 6.63354503372464

H 2.72558341565563 14.28943443399927 7.33263696066783

H 3.74473610479140 15.50246031171920 6.52972538277739

H 4.45053556784153 13.95968882408232 7.09139253635586

B 7.15632796007651 11.40246674699284 8.51237105487558

H 7.87792651579616 11.14121665546069 9.43909268205333

B 8.66951039388672 10.25579036532953 6.77064544937097

H 9.51377587845298 9.97998748401585 7.58184092474175

B 3.04527761593786 10.76227319868011 3.27968350221577

H 2.09285606192517 10.63363197776553 2.57456298278531

# **Supplementary Fig. S7. The PLQY variation of Ce-1 powder in the ambient environment.**

Sample storage condition: Temperature, 295 K ~ 303 K. Relative humidity, 30% ~ 60%.

# **Supplementary Fig. S8. Ultraviolet photoelectron** **spectroscopy of Ce-1.**

Based on the UPS spectrum and the UV absorption spectrum, the energy of frontier molecular orbitals of **Ce-1** are calculated as follows:

E_HOMO_ = -(hν-E_B_+E_A_) = -(21.22 eV-18.36 eV+3.36 eV) ≈ -6.2 eV,

E_LUMO_ = E_HOMO_+hc/λ = -6.2 eV +1240/400 eV ≈ -3.1 eV

# **Supplementary Fig. S9. Electroluminescence spectrum (black line) of device D1.**

The photoluminescence spectrum (red line) of BCPO:**Ce-1** (10 wt%) film was also plotted for comparison. The film was excited with 280 nm light. The electroluminescence spectrum was measured at 100 cd m^-2^.

# **Supplementary Fig. S10. Anisotropic refractive indices and extinction coefficient of Ce-1 doped films.**

The refractive indices (**a,c**) and extinction coefficient (**b,d**) of BCPO:**Ce-1** (10 wt%) and TSPO1:CzSi:**Ce-1** (0.18:0.72:0.1 in weight ratio) film, respectively.

# **Supplementary Fig. S11. Photoluminescence and electroluminescence spectra of Ce-1 doped films.**

**a,b.** The photoluminescence (red line) and electroluminescence (black line) spectra of **Ce-1** doped into CzSi (**a**) and TSPO1 (**b**). The doping concentration is 10 wt%, and the electroluminescence spectra were measured at 100 cd m^-2^. The photoluminescence spectra were measured with an excitation light of 280 nm.

# **Supplementary Fig. S12. Device operation lifetimes.**

The initial luminance is around 100 cd m^−2^.

Device configuration:

A: ITO/MoO_3_ (2 nm)/CzSi:MoO_3_ (20 wt%, 30 nm)/CzSi (10 nm)/CzSi:TSPO1:Ce-1 (0.18:0.72:0.1 in weight ratio, 20 nm)/TSPO1 (10 nm)/BPhen (40 nm)/LiF (0.7 nm)/Al (100 nm).

B: ITO/MoO_3_ (2 nm)/CzSi:MoO_3_ (20 wt%, 30 nm)/CzSi (10 nm)/CzSi:TSPO1:TPEA (0.19:0.77:0.04 in weight ratio, 20 nm)/TSPO1 (10 nm)/BPhen (40 nm)/LiF (0.7 nm)/Al (100 nm).

C: ITO/MoO_3_ (2 nm)/mCP:MoO_3_ (20 wt%, 30 nm)/mCP (10 nm)/mCP:TPEA (4 wt%, 20 nm)/TPBi (50 nm)/LiF (0.7 nm)/Al (100 nm).

In the same device configuration, **Ce-1** exhibits better stability than TPEA. The LT_50_ of device A (147 s) is almost 2 times longer than that of device B (67 s). But the lifetimes of both devices A and B are still very short. When TSPO1:CzSi was replaced by mCP, the LT_50_ of device C (TPEA as the emitter) is 14 times longer than device B, reaching 961 s. Thus the instability of TSPO1 should be responsible for device degradation.

# Supplementary Table S1. Selected coordination bond lengths (Å) of Ce-1.

| Ce1-O1 | Ce1-N12 | Ce1-N2 | Ce1-N4 | Ce1-N8 | Ce1-N6 | Ce1-N14 | Ce1-N10 |
| --- | --- | --- | --- | --- | --- | --- | --- |
| 2.398(3) | 2.598(5) | 2.628(5) | 2.645(4) | 2.649(4) | 2.653(5) | 2.666(5) | 2.673(4) |
| Ce2-O2 | Ce2-N24 | Ce2-N20 | Ce2-N16 | Ce2-N22 | Ce2-N26 | Ce2-N18 | Ce2-N28 |
| 2.399(3) | 2.573(5) | 2.611(5) | 2.646(4) | 2.646(5) | 2.672(4) | 2.678(5) | 2.680(5) |

# Supplementary Table S2. The PLQYs of Ce-1 solutions.

| **Ce-1** solvent | DCM | | | THF | Toluene |
| --- | --- | --- | --- | --- | --- |
| Concentration | 10^-3^ M | 10^-4^ M | 10^-5^ M | 10^-3^ M | 10^-3^ M |
| PLQY (%) | ~100 | 93 | 48 | 57 | ~100 |

# Supplementary Table S3. Molecular orientation measurements of Ce-1 doped films.

| Doped films | Extinction coefficient ^a)^ | | *S* ^b)^ | *h*/(*h+ν*) ^c)^ |
| --- | --- | --- | --- | --- |
|  | $\text{k}_{\text{o}}^{\text{max}}$ | $\text{k}_{\text{e}}^{\text{max}}$ |  |  |
| BCPO:**Ce-1** | 0.209 | 0.196 | -0.0212 | 0.681 |
| TSPO1:CzSi:**Ce-1** | 0.317 | 0.499 | 0.1606 | 0.560 |

^a)^Deduced from VASE molecular orientation measurement, the theory and calculation method were reported in the literature.^1^; ^b)^Order parameters; ^c)^Horizontal dipole ratios.

**OLEDs optimization details**. The device optimization includes screening host material, varying the TSPO1:CzSi ratio, finding the best combination of HTL and ETL, adjusting the doping concentration and the thickness of the emission layer. The detailed device structure and performance of each OLEDs are listed as following and in **Supplementary Table S4**, respectively.

# Supplementary Table S4. The key parameters of OLEDs with Ce-1 as the emitter.

| Devices | V_on_^a)^  [V] | EQE_max_^b)^  [%] | CE_max_^c)^  [cd A^-1^] | L_max_^d)^  [cd m^-2^] | CIE^e)^  (x, y) |
| --- | --- | --- | --- | --- | --- |
| O1 | 4.8 | 9.6 | 6.2 | 177 | 0.147, 0.078 |
| O2 | 4.5 | 7.3 | 4.7 | 935 | 0.149, 0.077 |
| O3 | 5.0 | 10.9 | 8.1 | 369 | 0.144, 0.089 |
| O4 | 5.1 | 8.9 | 6.4 | 948 | 0.144, 0.087 |
| O5 | 4.6 | 9.0 | 6.3 | 623 | 0.144, 0.084 |
| O6 | 4.6 | 10.0 | 7.1 | 403 | 0.144, 0.086 |
| O7 | 4.6 | 10.5 | 7.2 | 838 | 0.145, 0.082 |
| O8 | 4.4 | 9.1 | 6.4 | 935 | 0.145, 0.085 |
| O9 | 6.4 | 1.1 | 1.7 | 478 | 0.296, 0.228 |
| O10 | 4.8 | 11.2 | 8.12 | 395 | 0.143, 0.087 |
| O11 | 3.8 | 13.7 | 9.9 | 726 | 0.144, 0.086 |
| **O12/D2^f)^** | **3.6** | **14.0** | **10.3** | **1008** | **0.146, 0.078** |
| O13 | 4.0 | 10.9 | 6.7 | 1005 | 0.148, 0.071 |
| O14 | 3.9 | 11.3 | 7.4 | 724 | 0.146, 0.078 |
| O15 | 4.2 | 10.7 | 7.5 | 1040 | 0.145, 0.083 |
| O16 | 4.0 | 10.7 | 7.4 | 1020 | 0.146, 0.083 |
| O17 | 4.8 | 10.5 | 9.4 | 777 | 0.146, 0.109 |
| O18 | 4.5 | 11.9 | 11.2 | 954 | 0.142, 0.108 |
| O19 | 4.2 | 13.5 | 12.0 | 1184 | 0.143, 0.095 |
| O20 | 3.6 | 9.9 | 6.4 | 682 | 0.147, 0.076 |

^a)^Turn on voltage, is taken as a reference point at which the luminance is 1 cd m^-2^; ^b)^Maximum EQE; ^c)^Maximum current efficiency; ^d)^Maximum luminance; ^e)^Coordinates at 100 cd m^-2^. ^f)^Champion device.

1. Screening of the host material

**O1**: ITO/MoO_3_ (2 nm)/CzSi:MoO_3_ (20 wt%, 10 nm)/CzSi (30 nm)/TSPO1:**Ce-1** (10 wt%, 20 nm)/TSPO1 (10 nm)/TPBi (40 nm)/LiF (0.7 nm)/Al (100 nm)

**O2**:ITO/MoO_3_ (2 nm)/CzSi:MoO_3_ (20 wt%, 10 nm)/CzSi (30 nm)/CzSi:**Ce-1** (10 wt%, 20 nm)/TSPO1 (10 nm)/TPBi (40 nm)/LiF (0.7 nm)/Al (100 nm)

**O3**:ITO/MoO_3_ (2 nm)/CzSi:MoO_3_ (20 wt%, 10 nm)/CzSi (30 nm)/TSPO1:CzSi:**Ce-1** (0.45:0.45:0.1 in weight ratio, 20 nm)/TSPO1 (10 nm)/TPBi (40 nm)/LiF (0.7 nm)/Al (100 nm)

Different host materials TSPO1, CzSi and TSPO1:CzSi (1:1 in weight ratio) were tested in devices **O1-O3**. It’s found that the device **O3** shows the highest EQE of 10.9% (**Supplementary Table S3**). Therefore the TSPO1:CzSi co-host system was selected for further optimization.

1. Optimization of the TSPO1:CzSi ratio

**O4**: ITO/MoO_3_ (2 nm)/CzSi:MoO_3_ (20 wt%, 10 nm)/CzSi (30 nm)/TSPO1:CzSi:**Ce-1** (0.63:0.27:0.1 in weight ratio, 20 nm)/TSPO1 (10 nm)/TPBi (40 nm)/LiF/Al

**O5**: ITO/MoO_3_ (2 nm)/CzSi:MoO_3_ (20 wt%, 10 nm)/CzSi (30 nm)/TSPO1:CzSi:**Ce-1** (0.54:0.36:0.1 in weight ratio, 20 nm)/TSPO1 (10 nm)/TPBi (40 nm)/LiF/Al

**O6**: ITO/MoO_3_ (2 nm)/CzSi:MoO_3_ (20 wt%, 10 nm)/CzSi (30 nm)/TSPO1:CzSi:**Ce-1** (0.27:0.63:0.1 in weight ratio, 20 nm)/TSPO1 (10 nm)/TPBi (40 nm)/LiF/Al

**O7**: ITO/MoO_3_ (2 nm)/CzSi:MoO_3_ (20 wt%, 10 nm)/CzSi (30 nm)/TSPO1:CzSi:**Ce-1** (0.18:0.72:0.1 in weight ratio, 20 nm)/TSPO1 (10 nm)/TPBi (40 nm)/LiF/Al

**O8**: ITO/MoO_3_ (2 nm)/CzSi:MoO_3_ (20 wt%, 10 nm)/CzSi (30 nm)/TSPO1:CzSi:**Ce-1** (0.09:0.81:0.1 in weight ratio, 20 nm)/TSPO1 (10 nm)/TPBi (40 nm)/LiF/Al

Different host blending ratios varying from 0.63:0.27 to 0.09:0.81 (TSPO1:CzSi) were designed in devices **O4-8**. It’s found that the device **O7** exhibits the best EQE of 10.5% with the TSPO1:CzSi ratio of 0.18:0.72. Thus further optimization is based on this ratio.

1. Optimization of the HTL & ETL

**O9**:ITO/MoO_3_ (2 nm)/4,4′-cyclohexylidenebis[N,N-bis(4-methylphenyl)benzenamine] (TAPC, 30 nm)/CzSi (10 nm)/TSPO1:CzSi:**Ce-1** (0.18:0.72:0.1 in weight ratio, 20 nm)/TSPO1 (10 nm)/TPBi (40 nm)/LiF/Al

**O10**: ITO/MoO_3_ (2 nm)/CzSi:MoO_3_ (20 wt%, 30 nm)/CzSi (10 nm)/TSPO1:CzSi:**Ce-1** (0.18:0.72:0.1 in weight ratio, 20 nm)/TSPO1 (10 nm)/TPBi (40 nm)/LiF/Al

**O11**: ITO/MoO_3_ (2 nm)/CzSi:MoO_3_ (20 wt%, 30 nm)/CzSi (10 nm)/TSPO1:CzSi:**Ce-1** (0.18:0.72:0.1 in weight ratio, 20 nm)/TSPO1 (10 nm)/1,3,5-tri[(3-pyridyl)-phen-3-yl]benzene (TmPyPB, 40 nm)/LiF/Al

**O12/D2**: ITO/MoO_3_ (2 nm)/CzSi:MoO_3_ (20 wt%, 30 nm)/CzSi (10 nm)/TSPO1:CzSi:**Ce-1** (0.18:0.72:0.1 in weight ratio, 20 nm)/TSPO1 (10 nm)/Bphen (40 nm)/LiF/Al

**O13**: ITO/MoO_3_ (2 nm)/CzSi:MoO_3_ (20 wt%, 30 nm)/CzSi (10 nm)/TSPO1:CzSi:**Ce-1** (0.18:0.72:0.1 in weight ratio, 20 nm)/TSPO1 (10 nm)/Bphen (30 nm)/LiF/Al

In this section, the thickness of p-doping HTL (**O7** vs. **O10**), HTL materials (**O9** vs. **O10**), ETL materials (**O10** vs. **O11** vs. **O12**), and the thickness of ETL (**O12** vs. **O13**) are compared. It’s found that the device **O12/D2** is the best combination.

1. Optimization of the doping concentration

**O14**: ITO/MoO_3_ (2 nm)/CzSi:MoO_3_ (20 wt%, 30 nm)/CzSi (10 nm)/TSPO1:CzSi:**Ce-1** (0.19:0.76:0.05 in weight ratio, 20 nm)/TSPO1 (10 nm)/Bphen (40 nm)/LiF/Al

**O12/D2**: ITO/MoO_3_ (2 nm)/CzSi:MoO_3_ (20 wt%, 30 nm)/CzSi (10 nm)/TSPO1:CzSi:**Ce-1** (0.18:0.72:0.1 in weight ratio, 20 nm)/TSPO1 (10 nm)/Bphen (40 nm)/LiF/Al

**O15**: ITO/MoO_3_ (2 nm)/CzSi:MoO_3_ (20 wt%, 30 nm)/CzSi (10 nm)/TSPO1:CzSi:**Ce-1** (0.17:0.68:0.15 in weight ratio, 20 nm)/TSPO1 (10 nm)/Bphen (40 nm)/LiF/Al

**O16**: ITO/MoO_3_ (2 nm)/CzSi:MoO_3_ (20 wt%, 30 nm)/CzSi (10 nm)/TSPO1:CzSi:**Ce-1** (0.16:0.64:0.2 in weight ratio, 20 nm)/TSPO1 (10 nm)/Bphen (40 nm)/LiF/Al

**O17**: ITO/MoO_3_ (2 nm)/CzSi:MoO_3_ (20 wt%, 30 nm)/CzSi (10 nm)/TSPO1:CzSi:**Ce-1** (0.12:0.48:0.4 in weight ratio, 20 nm)/TSPO1 (10 nm)/Bphen(40 nm)/LiF/Al

The doping concentrations of **Ce-1** varying among 5%-40% are tested in devices **O12** and **O14-17**, the best concentration is found to be 10 wt% in **O12/D2**.

1. Optimization of the thickness of emission layer

**O18**: ITO/MoO_3_ (2 nm)/CzSi:MoO_3_ (20 wt%, 30 nm)/CzSi (10 nm)/TSPO1:CzSi:**Ce-1** (0.18:0.72:0.1 in weight ratio, 40 nm)/TSPO1 (10 nm)/Bphen (40 nm)/LiF/Al

**O19**: ITO/MoO_3_ (2 nm)/CzSi:MoO_3_ (20 wt%, 30 nm)/CzSi (10 nm)/TSPO1:CzSi:**Ce-1** (0.18:0.72:0.1 in weight ratio, 30 nm)/TSPO1 (10 nm)/Bphen (40 nm)/LiF/Al

**O12/D2**: ITO/MoO_3_ (2 nm)/CzSi:MoO_3_ (20 wt%, 30 nm)/CzSi (10 nm)/TSPO1:CzSi:**Ce-1** (0.18:0.72:0.1 in weight ratio, 20 nm)/TSPO1 (10 nm)/Bphen (40 nm)/LiF/Al

**O20**: ITO/MoO_3_ (2 nm)/CzSi:MoO_3_ (20 wt%, 30 nm)/CzSi (10 nm)/TSPO1:CzSi:**Ce-1** (0.18:0.72:0.1 in weight ratio, 10 nm)/TSPO1 (10 nm)/Bphen (40 nm)/LiF/Al

The thickness of EML varying among 10-40 nm are tested in devices **O12** and **O18-20**, the best thickness is found to be 20 nm in **O12/D2**.

# Supplementary Table S5. The summary of deep-blue OLEDs.

| Emitter | CIE(x, y) | EQE_max_  (%) | L_max_  (cd m^-2^) | Device lifetime (s)  @ 100 cd m^-2^ | References |
| --- | --- | --- | --- | --- | --- |
| Pt complex | 0.148, 0.079 | 24.8 | ~1000 | No report | ^2^ |
| Ir complex | 0.154,0.052 | 13.4 | ~300 | No report | ^3^ |
| TADF | 0.15, 0.06 | 21.5 | 1131 | No report | ^4^ |
| Perovskite | 0.094, 0.184 | 9.5 | ~700 | 250 | ^5^ |
| Ce complex | 0.146, 0.078 | 12.4 | 1008 | 147 | This work |

# References

1 Li, X. Y. *et al.* Deep blue phosphorescent organic light-emitting diodes with CIEy value of 0.11 and external quantum efficiency up to 22.5%. *Advanced Materials* **30**, 1705005, (2018).

2 Fleetham, T. *et al.* Efficient “pure” blue OLEDs employing tetradentate Pt complexes with a narrow spectral bandwidth. *Advanced Materials* **26**, 7116-7121, (2014).

3 Pal, A. K. *et al.* High-efficiency deep-blue-emitting organic light-emitting diodes based on iridium(iii) carbene complexes. *Advanced Materials* **30**, 1804231, (2018).

4 Ahn, D. H. *et al.* Highly efficient blue thermally activated delayed fluorescence emitters based on symmetrical and rigid oxygen-bridged boron acceptors. *Nature Photonics* **13**, 540-546, (2019).

5 Liu, Y. *et al.* Efficient blue light-emitting diodes based on quantum-confined bromide perovskite nanostructures. *Nature Photonics* **13**, 760-764, (2019).
